# Supplementary material for: Temporal proteomic analysis of HIV infection reveals remodelling of the host phosphoproteome by lentiviral Vif variants
Source: eLife. 2016 Sep 30;5:e18296. doi: 10.7554/eLife.18296 (PMC5085607; doi:10.7554/eLife.18296)
Supplement: Supplementary file 1. — Studies cited: Araínga et al., 2015; Haverland et al., 2014; Navare et al., 2012; Kraft-Terry et al., 2011; Rasheed et al., 2009; Chan et al., 2007; Chan et al., 2009; Ringrose et al., 2008; Pathak et al., 2009. DOI: http://dx.doi.org/10.7554/eLife.18296.027 [file elife-18296-supp1.docx]

## Supplementary file 1. Previous proteomic studies of HIV-infected cells

| Study | Proteomic analysis | Experimental design |
| --- | --- | --- |
| (Chan et al., 2007) | Whole cell proteomic analysis using an AMT tag database and trypsin catalysed 16O/18O exchange – 3,225 host proteins | CEMx174 cells infected *in vitro* at 2x TCID50 for 36 h with LAI strain of HIV-1 |
| (Ringrose et al., 2008) | Whole cell proteomic analysis using 2D gel-based quantitation – 1,921 protein spots | PM1 cells infected at high MOI for 42 h or low MOI for 7-10 days (“peak infection”) with LAI strain of HIV-1 |
| (Chan et al., 2009) | Whole cell proteomic analysis using label-free quantitation – 1,146 cellular proteins | Primary CD4^+^ T-cells infected at 2x TCID50 for multiple timepoints up to 48 h with LAI strain of HIV-1 |
| (Pathak et al., 2009) | Whole cell proteomic analysis using SILAC – 651 proteins | THP cells chronically infected with IIIB strain of HIV-1 |
| (Rasheed et al., 2009) | Whole cell proteomic analysis using 2D gel-based quantitation – denominator not stated | RH9 cells infected at an MOI of 1 for various timepoints up to 96 days with X4-tropic HIV-1 (no further details) |

| (Kraft-Terry et al., 2011) | Whole cell proteomic analysis using pulsed-SILAC – denominator not stated | Primary monocyte-derived macrophages infected at an MOI of 0.1 for multiple timepoints up to 7 days with ADA strain of HIV-1 |
| --- | --- | --- |
| (Navare et al., 2012) | Whole cell proteomic analysis using iTRAQ – 2,847 proteins | SupT1 cells infected at an MOI of 2.5 for 4, 8 and 20 h with LAI strain of HIV-1 |
| (Haverland et al., 2014) | Whole cell proteomic analysis using SWATH-MS – 3,608 proteins | Primary monocyte-derived macrophages infected at an MOI of 1 for 5 days with ADA strain of HIV-1 |
| (Arainga et al., 2015) | Whole cell proteomic analysis using SWATH-MS – denominator not stated | Primary monocyte-derived macrophages infected at an MOI of 0.1 for 7 days with ADA strain of HIV-1 |
